# Effect of Chinese herbal medicine on primary dysmenorrhea: A protocol for a systematic review and meta-analysis

**DOI:** 10.1097/MD.0000000000017191

**Published:** 2019-09-20

**Authors:** Lu Xu, Tian Xie, Tao Shen, Tianfeng Zhang

**Affiliations:** a School of Basic Medical Sciences, Chengdu University of Traditional Chinese Medicine, Chengdu, Sichuan; b Shenzhen Hospital of Guangzhou University of Chinese Medicine, Shenzhen, Guangdong, China.

**Keywords:** Chinese herbal medicine, primary dysmenorrhea, protocol, systematic review

## Abstract

**Background:**

Primary dysmenorrhea (PD), the most common gynecological diseases, seriously affects women's life and work; however, without more effective treatment. Chinese herbal medicine (CHM) has been widely used for relieving dysmenorrheic pain in patients with PD. To assess the effectiveness and safety of CHM in patients with PD, a systematic review and meta-analysis of current published evidence regarding CHM as treatment for PD would be conducted in this study.

**Methods:**

Literatures related to CHM for PD from the establishment of the database to June 2019 will be retrieved from the following databases: MEDLINE, EMBACE, Wed of Science and Cochrane Central Register of Controlled Trials, Chinese Biomedical Literature Database, Chinese National Knowledge Infrastructure, and Wanfang Database. There are no language restrictions for retrieving literature. Eligible randomized clinical trials (RCTs) evaluating the effectiveness and safety of CHM in PD patients will be put in the study including outcomes of pain intensity, clinical effectiveness rate, quality of life, and adverse events. By scanning the titles, abstracts and full texts, 2 reviewers will independently select studies, extract data, and assess the quality of study. Meta-analysis of RCTs will be conducted using Review Manager 5.1 software. The results will be presented as risk ratio for dichotomous data, and standardized or weighted mean difference for continuous data.

**Result:**

This study will provide high-quality available evidence for the treatment of PD with CHM based on pain, clinical efficacy, quality of life, and adverse events.

**Conclusion:**

The systematic review willto evaluate the efficacy of CHM in treating PD and provide evidence for clinicians.

**PROSPERO registration number:**

CRD42019121185.

## Abstract 2

Supplemental Digital Content is available in the text

## 1 Introduction

Dysmenorrhea is a common gynecological disease characterized by abdominal pain before or during menstruation.^[1]^ Dysmenorrhea usually accompanied by nausea, fatigue, headache, irritability, dizziness, vomiting, and diarrhea.^[2]^ Dysmenorrhea is divided into primary dysmenorrhea (PD) and secondary dysmenorrhea (SD).^[3]^ PD is defined as the absence of organic pathologic changes in reproductive organs which occurs shortly after menarche, while SD is defined as menstrual pain caused by organic diseases of the genital system such as adenomyosis, uterine malformation, and pelvic inflammatory disease.^[4]^

The prevalence of dysmenorrhea seems to vary around the world, from 50% to 90%,^[5]^ 80% in Western Australia, 60% in Canada,^[6]^ while 48.4% in Mexico.^[7]^ In a number of studies of young women, absenteeism rates for PD ranged from 34% to 50%.^[8]^ About 10% of the patients with PD are so severe that they are unable to work for 1 to 3 days a month. In the United States, the economic losses caused by absence due to PD are about 600 million working hours and over 2 billion dollars per year.^[9]^ In addition, PD patients who still insist on working could easily lead to decreased productivity, increased possibility of accidents, and decreased work quality.

Currently, it has been recognized that increased prostaglandin (PG) is the main pathogenesis of PD.^[10]^ PG could cause uterine smooth muscle contraction and spasm, resulting in decreased uterine blood flow, and at the same time increase the sensitivity of its peripheral nerves to pain, resulting in dysmenorrhea. In addition to PG, other factors may also be associated with PD.^[11]^ Interleukin could cause dysmenorrhea by constricting the smooth muscles of the uterus.^[12]^ In patients with dysmenorrhea, the content of oxytocin (OT) is much higher than that in women without dysmenorrhea. OT can directly act on uterine muscle cells to cause uterine contraction, and stimulate the release of PGs by endometrial cells at the same time, causing and aggravating dysmenorrhea.^[13]^ Researches have reported that mood, exercise, eating habits, and environment are correlated with dysmenorrhea.^[14]^

The first line of therapy for PD is nonsteroidal anti-inflammatory drugs (NSAIDs).^[15]^ 17% to 95% (mean 67%) of women relieve pain with NSAIDs.^[16]^ At present, the main side effects of NSAIDs are the renal disturbances, gastrointestinal complications, and cardiovascular events.^[17]^ Oral contraceptives (OCs) are also recommended for the treatment of PD, which can relieve pain in up to 70% to 80% of the patients.^[18]^ However, OCs could cause nausea, vomiting, weight gain, or vaginal bleeding. Therefore, more and more patients with PD are seeking alternative therapies to relieve the discomfort caused by menstruation.

TCM has treated PD for more than 2000 years and its featured therapies include Chinese herbal medicine (CHM), acupuncture, moxibustion, massage, cupping, qigong, food therapy, and so on. The effectiveness of acupuncture,^[19,20]^ moxibustion,^[21]^ massage^[22]^ and acupoint stimulation^[23]^ and massage in treating PD have been reviewed. According to the guidelines for gynecological practice in Japan, by the Japan Society of Obstetrics and Gynecology and Japan Association of Obstetricians and Gynecologists (2011 edition), traditional Chinese medicine (TCM) could be used for PD.^[24]^ A quite number of clinical trials have shown that CHM is beneficial to the treatment of PD. Daily et al provided evidence for the effectiveness of Ginger in the treatment of PD.^[25]^ Jaafarpour et al found that Cinnamon markedly lighten the severity and duration of menstrual pain.^[26]^ Rehman et al confirmed Rheum Emodi is able to relieve the symptoms of PD.^[27]^ Sun et al indicated that Guizhi Fuling capsule possessed a remarkable spasmolytic effect on uterine tetanic contraction through in vivo and in vitro experiments.^[28]^ Ding et al found that Shaoyao Gancao decoction had obvious analgesic effect on dysmenorrhea model mice.^[29]^ Huang et al verified that Shaofu Zhuyu decoction in the treatment of Cold – Stagnation and Blood – Stasis PD in rats, the mechanism is closely related to the regulation of mitogen-activated protein kinase pathway.^[30]^

In recent years, there have been a few systematic reviews on specific TCM prescriptions for treating PD. For instance, a systematic review and meta-analysis showed that Shaofu Zhuyu decoction was superior to the conventional drug group in the treatment of PD.^[31]^ Another systematic review and meta-analysis provide evidence for the superiority of Danggui Shaoyao powder in the treatment of PD.^[32]^ However, the efficacy and safety of CHM in treating PD are still unclear due to the large number of TCM prescriptions. The purpose of this study is to systematically review the available literatures on the therapeutic effect of CHM on PD.

A related systematic review has been published on the topic. In 2007, Zhu systematic review provided evidence for the treatment of PD with CHM, proving that CHM can alleviate menstrual pain.^[33]^ In the past 10 years, there have been many new reports on the randomized controlled trials (RCTs) of CHM in treating PD. Therefore, we believe it is necessary to conduct a new systematic review to evaluate the efficacy of CHM in treating PD and provide evidence for clinicians.

## 2 Methods

### 2.1 Registration

This study has been registered in the International prospective register of systematic reviews with the registration number of CRD42019121185. This protocol adheres to the preferred reporting items for systematic reviews and meta-analyses protocols 2015.^[34]^

### 2.2 Types of study

We will put all RCTs related to CHM for PD into the study of the effectiveness of CHM for PD.

### 2.3 Participants

This study will employ the diagnostic standards of the Clinical Guideline of PD by the Society of Obstetricians and Gynecologists of Canada.^[35]^ SD, such as endometriosis, uterine fibroids, and adenomyosis, was not taken in consideration in this study.

### 2.4 Interventions

Interventions of the including trials will be oral administration of CHM, which can be a decoction, granules, or CHM with other modern dosage forms. The dosage and treatment course are not limited. The intervention of CHM with traditional western medicine will make no difference.

### 2.5 Comparisons

The control group will include placebo, blank control, and conventional medicine (such as NSAIDs). If CHM is combined with western medicine (for instance CHM is administrated with the NSAIDs), the usage of western medicine in the control group should be consistent with that in the experimental group.

### 2.6 Outcome

The primary outcomes include:

(1) Pain: a reduction in pain (ie, menstrual pain) that occurs only during the intervention or as a result of the intervention, measured by a visual analog scale, other validated scales, or as a dichotomous outcome.
(2) Clinical effectiveness rate: an overall relief on symptoms (other menstruation-related symptoms) that occurs only during the intervention or occurred as a result of the intervention, measured by changes in dysmenorrhea symptoms and treatment effectiveness, and either self-reported, observed, or reported by other similar measures.

The secondary outcomes include:

(1) Quality of life measured with valid questionnaires.
(2) Adverse events.

### 2.7 Data sources

The databases reviewed to collect RCTS related to CHM for PD will be as follows: 4 English literature databases, which are Medline, Embase, Wed of Science and Cochrane Library, and 3 Chinese literature databases, which are Chinese Biomedical Literature Database, Chinese National Knowledge Infrastructure, and Wanfang Database. There is no limit by language or publication status. Additional resources, such as relevant meeting minutes and qualified research references, will also be searched manually. File 1 shows the strategy for searching the database; http://links.lww.com/MD/D236.

### 2.8 Data selection

**Figure 1:**
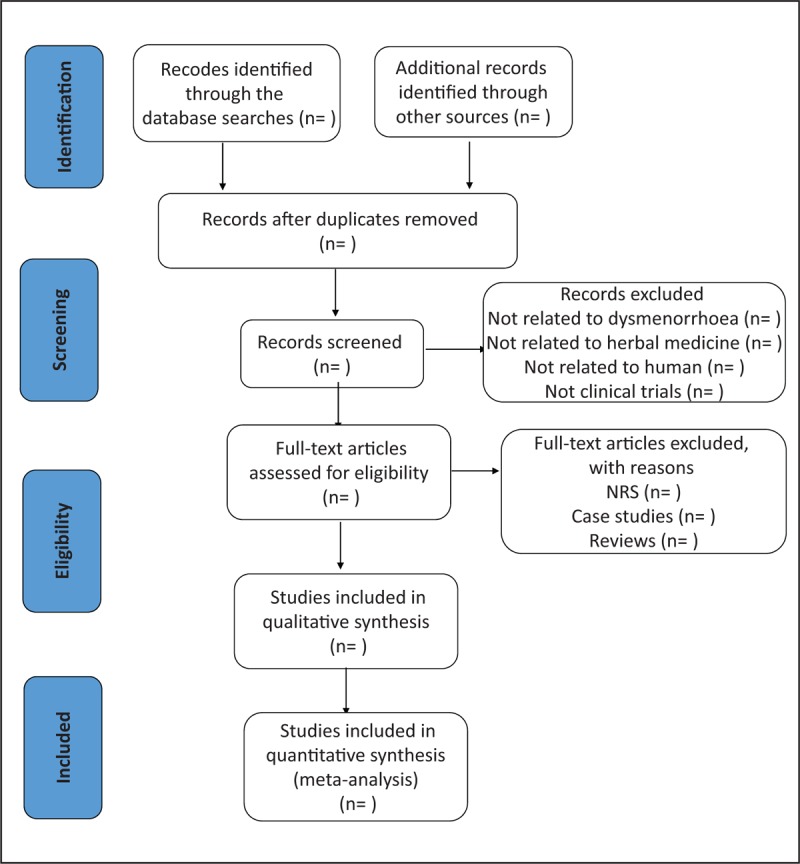
Flow diagram of study selection process.

Two reviewers independently run all literature search strategies, and records retrieved from the database are exported to the Endnote. According to the inclusion criteria, the titles and abstracts of references were determined by scanning literature retrieval, and irrelevant studies were excluded. Then the full text will be screened to determine eligible studies. Any differences between the reviewers will be resolved through discussion with a third reviewer. When the information is insufficient or unclear, we will contact the corresponding author via email or phone to ask for more information or clarification. Data extraction items included: first author, year of publication, diagnostic information, course of disease, sample size, age, intervention details, control and outcome, treatment time, follow-up time, and adverse events. The flow chart is shown in Figure 1.

### 2.9 Quality assessment

Bias risk was assessed using the Cochrane Handbook 5.1.0 bias risk assessment tool, including random sequence generation, allocation concealment, blinding of participants and personnel, blinding of outcome assessment, incomplete outcome data, selective reporting, and other sources of bias. This review will take L, U, and H to represent the assessment results: L means low risk of bias, U means unclear risk of bias, and H means high risk of bias. Differences will be resolved through discussion among all authors.

### 2.10 Data synthesis

Differences between the intervention group and the control group will be assessed. For continuous data, we will use the mean difference (MD) with 95% confidence intervals (CIs) to measure the therapeutic effect. We’re going to convert other forms of data into MDS. The standard MD of 95% CIs will be used for the outcome variables of different scales. We’re going to convert the other binary data into relative risk (RR) values. For binary data, we represent the processing effect as RRs with 95% CIs. All statistical analyses will be performed using the Cochrane Collaboration's software program Review Manager v. 5.3.5 for Windows. For research with insufficient data, we will contact relevant authors to obtain and verify data as far as possible.

### 2.11 Assessment of heterogeneity

Heterogeneity of the including studies will be analyzed by *χ*^2^ test and evaluated quantitatively by *I*^2^. If there is no significant heterogeneity between studies (*P* > .1 and *I*^2^ ≤ 50%), a fixed-effect model is adopted for meta-analysis. If there is heterogeneity among studies (*P* < .1, *I*^2^ > 50%), the sources of heterogeneity will be further analyzed. After excluding the effect of obvious clinical heterogeneity, a randomized effect model will be used for meta-analysis. Obvious clinical heterogeneity was treated by subgroup analysis, meta-analyst sensitivity analysis, or descriptive analysis.

### 2.12 Subgroup analysis

If there are a plenty of subgroup studies, subgroup analysis will be analyzed to determine the heterogeneity between them. The subgroup analysis criteria are as follows:

(1) treatment time or dose of CHM;
(2) duration or severity of PD;
(3) syndrome differentiation;
(4) formulations.

### 2.13 Sensitivity analysis

Sensitivity analysis will be conducted to test the robustness of key decisions made during the review process. The main decision nodes include method quality, sample size, and the impact of missing data.

### 2.14 Reporting bias

Reporting bias, such as the publication deviation will be evaluated by a funnel chart. When there are more than 10 trials in the analysis. If the visual examination shows asymmetry, we will use the Egger method for exploratory analysis.

### 2.15 Ethics and dissemination

Ethical approval is not required for this study because we did not use data related to individual patient. In addition, the findings will be disseminated through peer-reviewed publications or presentations at conferences.

## 3 Discussion

PD is a common disease in women. In severe cases, it can affect patients’ life and work, bringing economic and social burdens. At present, the treatment of PD by western medicine mainly consists of analgesia, sedation, and spasmolysis. Although the pain can be postponed, there are many adverse reactions and the long-term effect is not good. More and more patients with PD are seeking alternative treatment. CHM has attracted more and more attentions in PD because of its small side effects. CHM has been used to treat PD for more than 2000 years. In recent years, CHM has been extensively used in the treatment of PD. Although there have been studied on the efficacy of individual formulations for dysmenorrhea, the relative differences between these formulations are still uncertain. This study will be a comprehensive review of CHM treatment and provide basis for clinical gynecologists to use CHM to treat PD.

## Author contributions

**Conceptualization:** Tao Shen.

**Formal analysis:** Lu Xu, Tian Xie.

**Funding acquisition:** Tao Shen.

**Investigation:** Lu Xu, Tian Xie.

**Methodology:** Lu Xu, Tian Xie.

**Project administration:** Tao Shen.

**Supervision:** Tianfeng Zhang.

**Validation:** Tianfeng Zhang.

**Writing – original draft:** Lu Xu.

**Writing – review & editing:** Tao Shen.

## Supplementary Material

Supplemental Digital Content
